# Antibiotics Usage and Avoidance in Germany and Poland: Attitudes and Knowledge of Patients, Physicians, and Pharmacists

**DOI:** 10.3390/antibiotics13121188

**Published:** 2024-12-06

**Authors:** Andrzej M. Fal, Ingrid Stelzmüller, Peter Kardos, Ludger Klimek, Ernest Kuchar, André Gessner

**Affiliations:** 1 Department of Allergy, Lung Diseases and Internal Medicine, National Institute of Medicine, Ministry of Interior, 02-507 Warsaw, Poland; 2 Faculty of Medicine, Collegium Medicum, Cardinal Stefan Wyszynski University, 02-507 Warsaw, Poland; 3 Private Practice for Pulmonology, Internal Medicine and Pneumology, 5020 Salzburg, Austria; praxis@drstelzmueller.at; 4 Lung Centre Frankfurt, Red Cross Maingau-Hospital, 60316 Frankfurt am Main, Germany; kardos@lungenzentrum-maingau.de; 5 Centre for Rhinology and Allergology, 65183 Wiesbaden, Germany; ludger.klimek@allergiezentrum.org; 6 Department of Pediatrics with Clinical Assessment Unit, Medical University of Warsaw, 02-091 Warsaw, Poland; ernest.kuchar@wum.edu.pl; 7 Institute for Clinical Microbiology and Hygiene, University Clinic Regensburg, 93053 Regensburg, Germany; andre.gessner@klinik.uni-regensburg.de

**Keywords:** antibiotics, antimicrobial resistance, antibiotic stewardship, infections, behavior

## Abstract

**Introduction:** Antimicrobial resistance poses a significant global health threat, partly due to the overprescription of antibiotics. Understanding prescribers’ behaviors and identifying knowledge gaps and misconceptions are essential for addressing antibiotic misuse and inappropriate use. **Methods:** Through online questionnaires, this study surveyed key stakeholders in outpatient antibiotic use in Germany (DE) and Poland (PL), including patients, physicians, and pharmacists. **Results:** Despite generally good knowledge about antibiotics, discrepancies exist between physicians’ perceptions and patients’ actual expectations regarding antibiotic prescriptions. Physicians often misjudge patients’ attitudes toward antibiotics, with many patients having a neutral stance. This study found a strong physician interest in non-antibiotic treatments and patient willingness to engage with information about antibiotics. **Conclusions:** Improved communication between healthcare providers and patients was identified as a potential measure for enhancing antimicrobial stewardship, with education on effective alternative treatments, such as symptomatic therapies, as a likely strategy to reduce antibiotic reliance.

## 1. Introduction

Antibiotic overuse and unjustified use contribute to antimicrobial resistance (AMR), undermining critical medical treatments across populations and countries [1,2]. The European Economic Area (EEA) saw a general 23% decline in antibiotic usage from 2011 to 2020 with most countries reporting decreases in antibiotic consumption for both the community sector and the hospital sector. In the community sector, which accounts for 90% of antibiotics consumption in the EEA, this decrease was generally larger than in the hospital sector [1]. Despite this development, AMR has risen since 2011 and so has the prescription of broad-spectrum antibiotics [1]. Infections with antibiotic-resistant bacteria cause around 33,000 deaths annually in the EEA [1]. Globally, AMR was responsible for 1.27 million deaths in 2019, with projections suggesting it could become the leading cause of mortality by 2050 [1,3]. The COVID-19 pandemic has further exacerbated AMR worldwide due to the unjustified use of antibiotics, particularly in low- and middle-income countries [4].

Antibiotic consumption differs significantly across European countries. Germany reports low antibiotic use, while Poland exhibits high usage rates [5]. In Germany, prescriptions have dropped by 21% from 2010 to 2018, aligning with broader European trends [6]. However, Germany still sees high prescription rates of reserve antibiotics and broad-spectrum antibiotics in outpatient care [7,8]. In Poland, ambulatory antibiotic use is higher than in Germany and ranks among Europe’s highest, with notable differences among its regions [5,9].

Considering the health risks associated with the inappropriate use of antibiotics, efforts were undertaken to identify the root causes of this practice. Studies have shown that many antibiotics are prescribed unnecessarily regarding underlying conditions and drug choices [10]. A Polish study revealed that antibiotics are often used for viral illnesses such as the common cold, sore throat, cough, and flu, where they are generally unnecessary [11]. Factors contributing to such unjustified use in Europe include a lack of public understanding, the ability to obtain antibiotics without a prescription, and the use of leftover medications [12]. Healthcare providers may be inclined to overprescribe antibiotics due to concerns about their patient relationship, patient demands, potential malpractice issues, insufficient education, and insufficient time to discuss proper treatment plans [12,13,14]. Moreover, second line reserve antibiotics are often used if patients report suspected allergies to antibiotics, especially to penicillins and other ß-lactam antibiotics [15,16,17].

Various strategies, such as public information campaigns, have been suggested to decrease the inappropriate use of antibiotics. For instance, following the European Antibiotic Awareness Day efforts, there was a decline in patients citing typical viral infections as reasons for taking antibiotics or expecting prescriptions [11]. Furthermore, educational initiatives can contribute to an increase in the number of antibiotic prescriptions that align with clinical guidelines [18]. In addition, if an antibiotic treatment is justified at all, second line reserve antibiotics should be avoided whenever possible and proper measures have to be taken to achieve this goal [19,20,21,22,23,24,25,26].

Supporting these outcomes, research from Poland indicates that information about careful antibiotic use given in the past year has positively altered doctors’ opinions and practices [27]. In everyday medical settings, doctors note that patients often want to leave with something that could potentially ameliorate their symptoms [28]. This expectation of patients makes delayed prescriptions an effective approach [28]. With this method, patients are encouraged to only fill their prescription for antibiotics when their symptoms worsen.

In summary, there is an urgent need to minimize the inappropriate use of antibiotics further. Concurrently, encouraging cases have illustrated that awareness-raising initiatives, like campaigns, can yield positive outcomes [11,18,27]. The consumption of antibiotics is significantly impacted by the behavioral patterns of three key groups—patients, physicians, and pharmacists. In this dynamic, the overall antibiotic use is shaped by patients requesting or demanding antibiotics and physicians prescribing them. However, another part of this dynamic is the pharmacist, who supports the correct usage of antibiotics. Pharmacists are the earliest points of contact and contribute significantly to the patient’s education, for example by recommending effective symptomatic treatments where antibiotics are without effect as is the case with the common cold.

Consequently, this survey was designed to assess the present attitudes, knowledge, and practices of patients, physicians (including general practitioners (GPs), pediatricians (PEDs), and ear–nose–throat (ENT) specialists), and pharmacists in Germany and Poland in the outpatient sector. The aim was to receive an overview of attitudes and knowledge regarding the use of antibiotics. Additionally, we aimed to determine areas for future intervention to curtail the unjustified prescription of antibiotics. In this context, Germany and Poland represent European nations with moderate and high levels of antibiotic consumption, respectively.

## 2. Results

**Figure 1:**
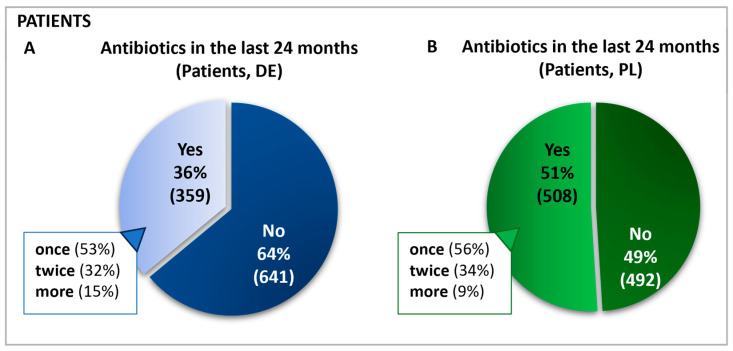
Frequency and percentage of antibiotic use in the past 24 months in Germany (**A**) and Poland (**B**). Questions: Patients: Have you taken antibiotics in the past 24 months? If yes: For how many ailments have you taken antibiotics in the last 24 months?

The survey was conducted in 2021 in Poland and Germany using self-administered online questionnaires to assess participants’ antibiotic use and avoidance behaviors. In Germany, 1000 patients, 181 physicians, and 142 pharmacists participated. In Poland, 1000 patients, 146 physicians, and 134 pharmacists participated. Of these 2000 patients, 867 reported having received antibiotics in the prior 24 months (Figure 1), with subsequent questions answered by this antibiotics-receiving subgroup. 

### 2.1. Frequency, Sources, and Reasons for the Use of Antibiotics

A total of 1000 patients in each country started the online survey and those who had been administered antibiotics in the previous 24 months completed all subsequent questions. Hence, the group labeled as “antibiotics receivers” represents 100% of the sample base regarding follow-up queries, and all further data are calculated relative to this cohort. This sample included n = 508 in Poland and n = 359 patients in Germany. For simplification, this group will be referred to as “patients”. In Germany and Poland, most patients reported taking antibiotics for only one condition in the last two years. Approximately one-third in each country had taken antibiotics for two different ailments (Figure 1).

As part of their specific survey section, physicians reported the average number of patients seen and antibiotic prescriptions written per week (Figure S1). Given that physicians’ prescribing habits vary according to their specialty and seasonally, the specialties were only presented when this was relevant for the interpretation. Physicians from Germany and Poland were each grouped by country. The data summarized in Table 1 indicate the number of physicians per specialty for each country. Overall, the findings reveal that between 110 (PL) and 178 (DE) patients are typically seen weekly in a medical practice. Over that same period, roughly 40% of these practices dispense between 1 and 10 antibiotic prescriptions (Figure S1). In Germany, 15%, and in Poland, 9% of patients received more than two antibiotic treatments.

Patients received antibiotics mostly from their doctor, either on prescription for immediate use (60% DE, 69% PL) or as a “precaution” (20% DE, 25% PL) (Table 2). The use as “precaution”, as stated by patients, could mean delayed prescription or precautious use in the case of medical interventions. Thus, in general, patients can be regarded as primarily compliant. The proportion of use as “precaution” was surprisingly high. However, here it has to be noted that patients reported from their memory without further specification of the circumstances.

**Figure 2:**
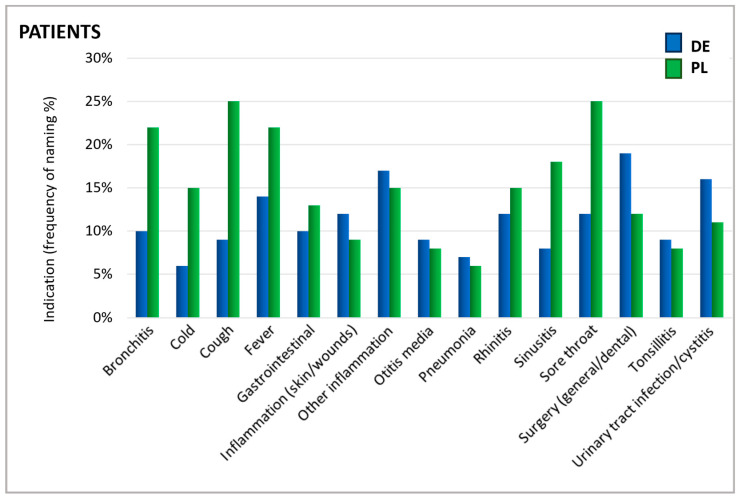
Indications for which antibiotics were taken. Question: Patients: What was the reason/were the reasons for taking antibiotics in the past 24 months (aided answer, multiple answers allowed)?

As shown in Figure 2, patients were asked for which cause they took an antibiotic. The reported indications showed a similar distribution in Germany and Poland. The most prominent reported uses were sore throat, fever, cough, bronchitis, inflammations, and surgical interventions/dental treatments (Figure 2).

**Figure 3:**
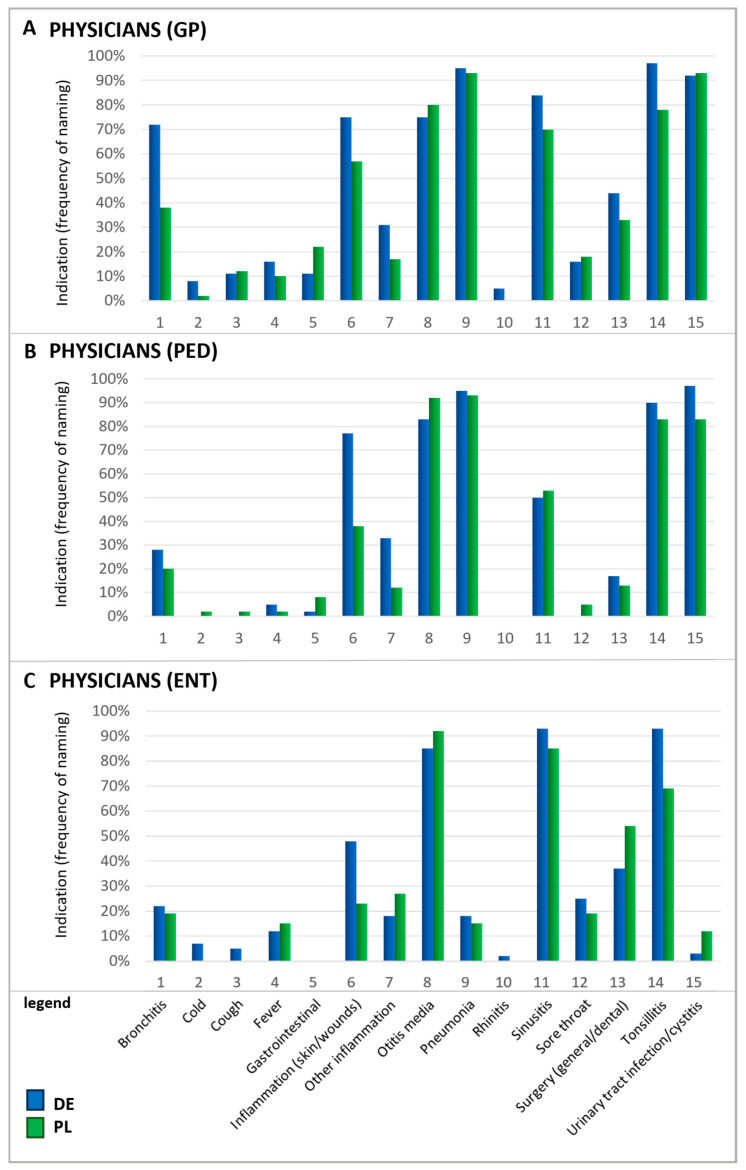
Indications for which antibiotics were prescribed by physicians. Question: Physicians: If you think about these prescriptions, for which indications do you prescribe antibiotics in general (multiple answers possible)? Please note that the results for ENTs in Poland are based on a small number of (n = 26). GP, general practitioners (**A**); PED, pediatricians (**B**); ENTs, ear–nose–throat specialists (**C**).

In particular, upper respiratory symptoms were more frequently cited as a reason for antibiotic use by Polish patients than by their German counterparts. Notably, for several conditions, there is a mismatch between the reasons patients believe antibiotics are needed and the reasons physicians give for prescribing them. For instance, rhinitis—referred to by its colloquial terms in German and Polish questionnaires—was one of the principal reasons patients in Poland named for using antibiotics. In contrast, physicians rarely identified this condition as a justification for antibiotic prescription (Figure 3). According to physicians, urinary tract infections (UTIs), otitis media, tonsillitis, and pneumonia were the most common ailments warranting antibiotics. Since ENT physicians do not typically treat urinary tract infections, they generally do not prescribe antibiotics for this indication in daily practice which explains the low percentage of ENT physicians prescribing antibiotics in this case.

Furthermore, physicians also mentioned acute bronchitis and sinusitis as relatively frequent reasons for prescribing antibiotics. It is important to recognize that acute respiratory infections are typically viral induced, so that they do not respond to antibiotic treatment and often resolve on their own [29,30]. A recent study estimated that nearly half of the antibiotic consumption in the outpatient sector is attributable to respiratory infections although viral infections are the root cause of 85–95% of acute bronchitis and cough cases and 70–80% of rhinosinusitis occurrences [30]. In direct comparison, patients’ beliefs about antibiotic usage and the physician’s judgment differ, indicating a knowledge gap between those two groups and the need for better-informed patients.

### 2.2. Knowledge and Expectations Regarding Antibiotics and Alternatives

**Figure 4:**
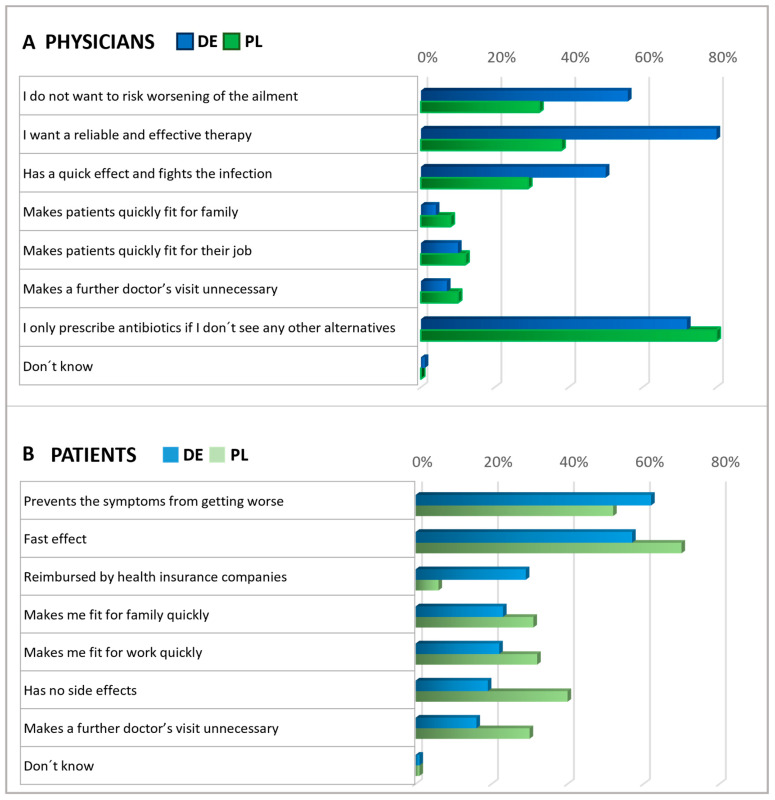
Physicians’ and Patients’ expectations of antibiotics. Question: Physicians (**A**): What statements do you think apply to antibiotics (multiple answers)? Patients (**B**): What expectations do you associate with antibiotics (multiple answers possible)? The agreement with the statements is displayed in % of all given answers per group (ranking).

**Figure 5:**
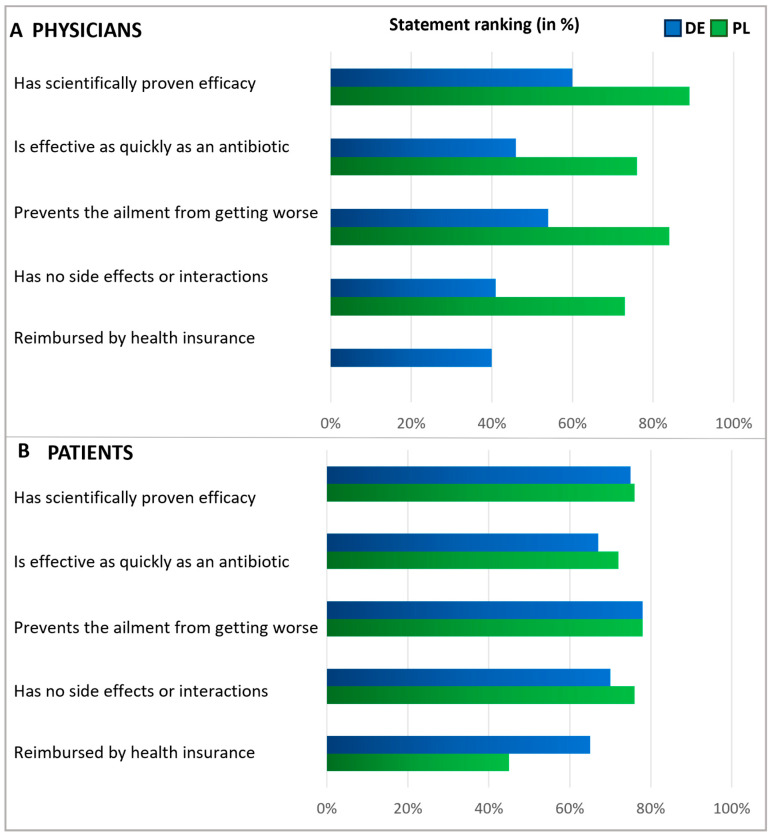
Expectations of an alternative treatment for antibiotics. Questions: Physicians (**A**): What requirements do you place on alternative products to antibiotics? Please answer on a scale of 1 to 5, where 1 means‚ it is not important at all and 5 ‘is very important.’ Patients (**B**): What requirements do you place on alternative treatments to antibiotics? Please answer on a scale of 1 to 5, where 1 means‚ it is not important at all and 5 ‘is very important.’ The graphic shows the grouped statements of 1 and 2 in %, according to the relative number of statements (ranking). Polish physicians were not asked about reimbursement.

The objective was to assess the knowledge and expectations regarding antibiotics (Table 3, Figure 4 and Figure 5). Patients and physicians were asked to indicate their level of agreement with specific statements about antibiotics. The percentage of respondents who agreed with each statement was calculated (Table 3). Physicians almost unanimously agreed that antibiotics are effective against bacteria but not viruses. In contrast, only about 40% of patients correctly answered that antibiotics are effective against bacteria, and 20% of patients incorrectly believed that antibiotics are effective against viruses. Interestingly, there was a marked difference between German and Polish patients regarding the awareness of side effects, with German patients being more aware of this issue. The knowledge about long-term side effects was less prevalent, with surprisingly few physicians being aware of long-term side effects (DE 23%, PL 14%).

Subsequently, patients and physicians were asked about their expectations for antibiotics (Figure 4). Among patients, the main expectations for antibiotics were rapid relief and the prevention of symptoms worsening. Roughly a third of participants in Germany anticipated that their health insurance would cover the cost of antibiotics, while in Poland, this expectation was not common among patients (6%), suggesting that insurance reimbursement was not a significant concern in Polish patient considerations at the time of the survey. Importantly, in both countries, antibiotics are only available by prescription.

In both countries, physicians commonly reported prescribing antibiotics only when they saw no other viable option (Figure 4). In Germany, doctors frequently expressed the expectation that antibiotics should be a reliable and effective treatment. However, Polish physicians agreed with this opinion to a considerably lesser extent, indicating a notable disparity between the two countries. About half of the physicians in Germany and one-third of the physicians in Poland shared the anticipation that antibiotics might lower the risk of worsening an ailment.

In a subsequent question, patients and physicians were presented with various statements to assess their expectations regarding alternatives to antibiotics, with respondents rating the significance of each statement on a scale of 1 to 5 (Figure 5). Answers rated as the top two choices were interpreted as agreement. Regarding expectations of alternatives to antibiotics, the majority within each subgroup emphasized the importance of treatments that prevent the ailment from worsening. Notably, fewer than half of the German physicians expected alternatives to be free from side effects, whereas most Polish physicians and patients shared this expectation. About half of the patients expected alternative treatments to be covered by health insurance, with a more significant proportion of German patients holding this expectation than Polish ones. In addition, but not shown here in detail, physicians were asked about their measures to secure an antibiotic prescription. The results revealed that the prescription of antibiotics usually relies on examining typical symptoms and is based on experience (Figure S2).

### 2.3. Expectations During a Doctor’s Consultation

**Figure 6:**
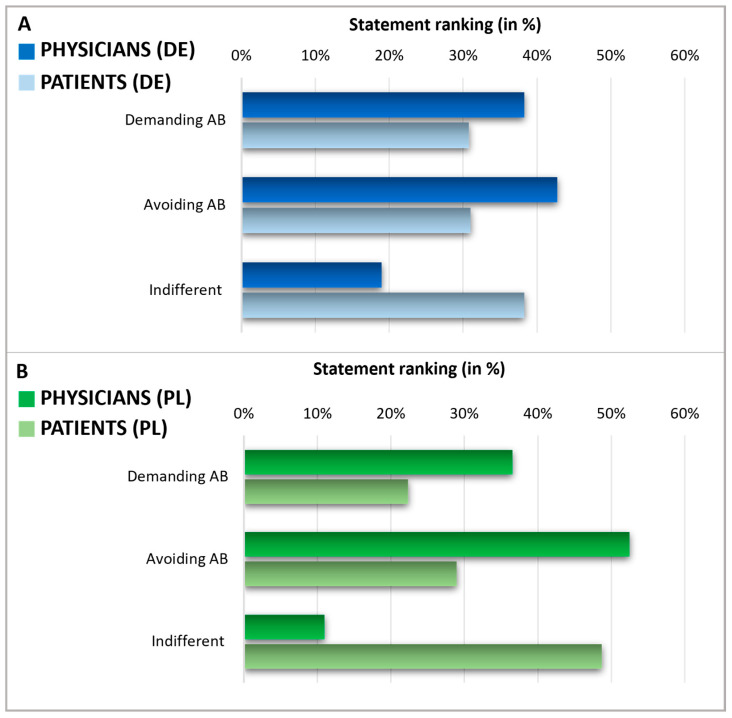
Expectations during a doctor’s consultation. Graphics comparing physicians and patients in Germany (**A**) and Poland (**B**). The displayed statement rankings show the grouped answers in %, indicating a discrepancy in the expectations of physicians and patients about demanding or avoiding AB. Questions: Patients: Suppose: You are at the doctor with complaints for which, from your point of view, the use of an antibiotic would be conceivable. As answers, 5 different statements were presented (2 of these counted as demanding AB, 2 of these counted as avoiding AB, 1 of these counted as indifferent). Multiple answers were possible and grouped into three categories. Consecutively, the proportion of demanding/avoiding/indifferent relative to the total number of answers was calculated and expressed in percentage. Physicians: Please indicate how the following scenarios occur proportionally in your daily practice—regardless of whether you prescribe antibiotics in these cases. Estimate the percentage share—the sum of the answers must add up to 100. Possible answers were scenarios with the patient demanding AB, avoiding AB, or being indifferent.

After assessing the knowledge and expectations regarding antibiotics, we analyzed patients’ expectations towards their doctors’ prescribing of antibiotics and physicians’ perceptions of these patient expectations in Germany and Poland (Figure 6). To do this, patients were queried about what they expected from their doctor when they considered using antibiotics. Physicians were asked to estimate their patients’ expectations regarding antibiotic prescriptions. Notably, in both countries, the self-reported percentage of patients indifferent to antibiotic prescription (38% in Germany, 49% in Poland) was higher than the percentage of patients who either tend to avoid antibiotics (31% in Germany, 29% in Poland) or those who demand them (31% in Germany, 22% in Poland). On the other hand, physicians assumed a much smaller segment of their patients were indifferent to antibiotic prescriptions (19% in Germany and 11% in Poland), thus considerably underestimating the level of patient indifference. As a result, physicians overestimated the factions of patients who avoided or demanded antibiotics. In Germany, physicians believed that 38% of patients would demand antibiotics and that 43% would prefer to avoid them. Polish physicians assumed that 37% of patients would demand antibiotics and 52% would prefer to avoid them.

### 2.4. Responsibility of Antibiotics Avoidance

**Figure 7:**
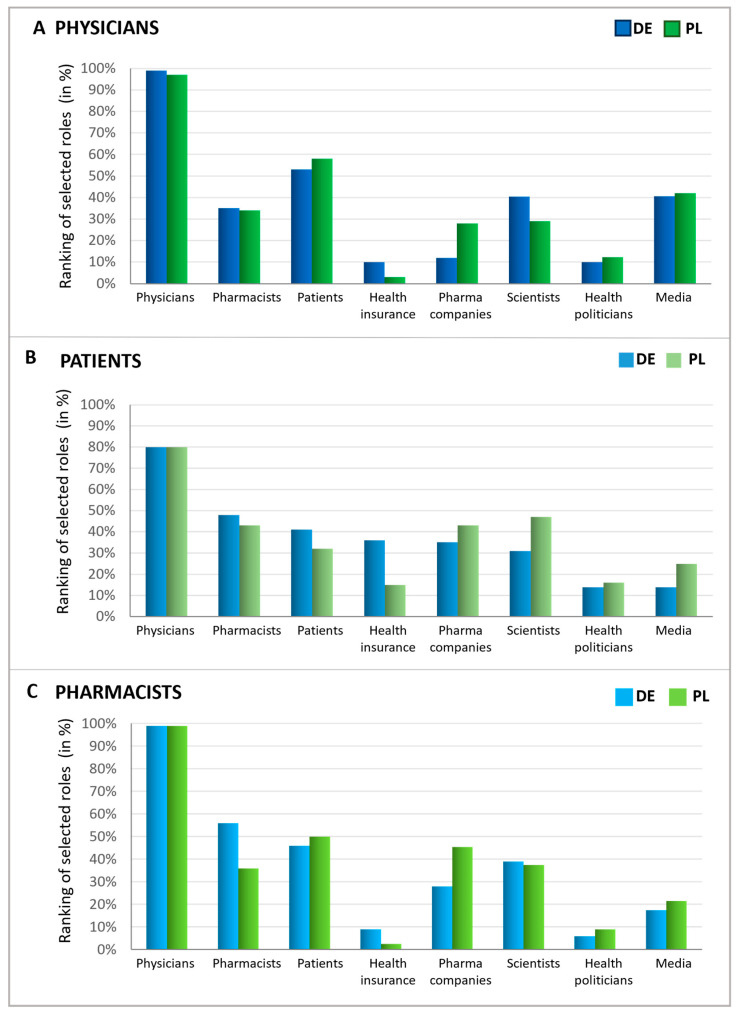
Responsibilities in antibiotics avoidance seen by different groups. Graphics show the possible influence of different players (function), assessed by physicians (**A**), patients (**B**), and pharmacists (**C**), ranked in % according to multiple entries. For all three groups, possible answers were physicians, pharmacists, patients, pharmaceutical companies, scientists, health politicians, media/journalists, health insurance funds, and none of these. The participants were asked to name the three most important out of the 8 groups or institutions.

Upon examining expectations during a doctor’s visit, the perceived responsibility of various groups for antibiotic stewardship was evaluated. To this end, patients, pharmacists, and physicians were queried about which groups or institutions have the greatest responsibility for avoiding the inappropriate use of antibiotics (Figure 7). The consensus across all respondent groups and countries indicates that physicians are widely viewed as having the primary responsibility for antibiotic stewardship. Intriguingly, patients also considered themselves to be responsible but slightly less responsible than pharmacists. On the other hand, physicians regard patients as having greater responsibility than pharmacists, whereas German pharmacists view themselves as more accountable than patients. Polish pharmacists expressed that they view patients as more responsible in this matter than themselves. These findings suggest that while doctors are seen as most responsible for antibiotic stewardship, there is recognition that pharmacists and patients also play significant roles in this effort.

Furthermore, other groups were also seen as responsible for avoiding the inappropriate use of antibiotics to varying extents. In all queried groups, over one-third identified scientists as key figures in combating the inappropriate use of antibiotics, which was the most significant recognition following the primary groups (patients, physicians, and pharmacists). Physicians particularly noted the role of the media, while patients pointed to health insurance funds and pharmaceutical companies. Similarly, pharmacists attributed a notable level of responsibility to pharmaceutical companies, comparable to that of scientists. Health politicians, however, were acknowledged as responsible by only a small fraction of individuals within each group.

### 2.5. Attitudes on Measures for Reducing the Use of Antibiotics

Subsequently, the attitudes of pharmacists toward discussing various antibiotics-related issues in interactions with patients were examined (Table 4). Notably, pharmacists typically prioritized topics directly related to antibiotic usage. These topics include the frequency and duration of use (93% of German pharmacists and 87% of Polish pharmacists) and advice on potential side effects (80% in Germany, 66% in Poland). Advice on drug interactions was considered less critical by Polish pharmacists (66%) compared with their German counterparts (89%). In contrast, issues indirectly associated with antibiotic use, such as the overarching reduction of antibiotics (44% in Germany, 45% in Poland) or alternative therapies (39% in Germany, 30% in Poland), were deemed less important for customer conversations by pharmacists from both countries.

Considering helpful measures to reduce the use of antibiotics, Polish pharmacists in particular saw the value in using rapid tests to detect bacterial or viral infections as a helpful strategy (81%). In comparison, German pharmacists agreed to a smaller extent (54%). The preferred measure among pharmacists in Germany was the availability of suitable alternative treatments (58%), while Polish pharmacists found this option less important (35%). German pharmacists also supported the idea of a consultation tool outlining common conditions with alternatives to antibiotics (52%). These findings suggest that German and Polish pharmacists take somewhat different approaches to antibiotic stewardship.

Physicians generally expressed a preference for rapid tests capable of distinguishing between bacterial and viral infections (Table 5). Polish physicians had a higher level of agreement with the utility of such tests (90%) in comparison with their German counterparts (77%), reflecting a similar pattern to that observed among pharmacists, with Polish respondents being more likely to consider rapid testing beneficial than their German colleagues. Other measures that were particularly supported included “Clinical studies demonstrating the suitability of non-antibiotic therapies” (76% in Germany, 86% in Poland), “Medical guidelines that include more alternative ingredients and therapies to antibiotics” (63% in Germany, 86% in Poland), and “Better informed patients” (73% in Germany, 82% in Poland). Interestingly, consistent with pharmacists’ views, physicians also deemed delayed prescriptions less effective than other measures (46% in Germany and 37% in Poland). Additionally, 70% of German physicians believed that improved compensation for time spent in patient counseling would be helpful, whereas only 33% of Polish physicians saw this as an important factor.

### 2.6. Information About Antibiotics and Willingness to Help Prevent Unjustified Use

**Figure 8:**
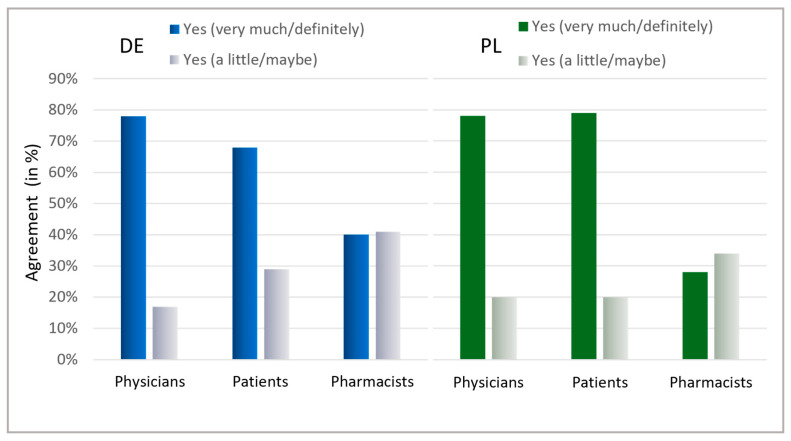
Interest in information about AB and willingness to avoid AB. Question: Patients: Is it important to you to be informed about antibiotics? Possible answers: Yes, very much; Yes, a little; No. Physicians: Would you personally like to contribute to further reducing antibiotic intake? Possible answers: Yes, definitely; Yes, maybe; No; I would like to, but do not see the possibility; Do not know. Pharmacists: Would you personally like to contribute in your pharmacy to further reduce the intake of antibiotics? Possible answers: Yes, definitely; Yes, maybe; No; I would like to, but do not see the possibility; Do not know. For physicians and pharmacists, the results for the answers “Yes, definitely” and “Yes, maybe” are presented here.

Ultimately, the patients’ interest in learning about antibiotics was assessed (Figure 8). A general interest exists within both countries, with a majority indicating they are highly interested (Germany: 68%, Poland: 79%). Notably, in Germany, 97%, and in Poland, 99% of patients displayed at least some interest in information on antibiotics. The willingness among physicians to contribute to antibiotic stewardship was also high across both countries, with 78% stating they are willing to contribute personally and another 17% (Germany) and 18% (Poland) considering the possibility. Surprisingly, pharmacists needed more confidence in their commitment, with only 40% in Germany and 28% in Poland expressing a clear willingness to contribute to reducing antibiotic usage. Nevertheless, the considerable percentage of “maybe” responses (Germany: 41%, Poland: 34%) could indicate that pharmacists are uncertain about their role and effectiveness in antibiotic stewardship.

## 3. Discussion

### 3.1. Current Challenges for Reduction of Antibiotics Use

While there are encouraging trends, certain conditions suggest that more work is needed to further reduce the inappropriate use of antibiotics. The most significant decline in antibiotic use in the EU/EEA was observed between 2019 and 2020, coinciding with actions taken against COVID-19 in 2020 [1]. Noticeably, the decrease in the prescription of antibiotics was particularly prominent for respiratory infections and young age groups [1]. It is highly likely that non-pharmaceutical interventions to mitigate the transmission of SARS-CoV-2 had an effect on the transmission of a larger set of infectious diseases. Furthermore, a decrease in the prescription of antibiotics was attributed to the reduced use of primary care services for milder infections [1]. Overall, this suggests that the decrease in respiratory illnesses other than COVID-19, as well as the reduced number of doctor visits, may account for the substantial reduction in antibiotic consumption during this period. Importantly, these observations were made for the situation in Europe [1,31,32]. On a worldwide scale, the COVID-19 pandemic has further exacerbated AMR due to the unjustified use of antibiotics, which was particularly prevalent in low- and middle-income countries [4].

There is considerable variability in antibiotic use between European countries and within regions in both outpatient and inpatient healthcare settings [1,7,9,33]. Differences are not only present between countries or regions but also between individual practices. For instance, a German study highlighted that a small subset of practices writes a disproportionately high rate of antibiotic prescriptions, which implies that targeting these practices could reduce overall national consumption [10].

Poland has one of the highest rates of antibiotic use in Europe, ranking fifth in consumption among 23 countries in 2016 [5]. This is largely driven by over-the-counter sales of furazidine, an antibiotic for uncomplicated urinary tract infections, which accounts for 15% of systemic antibiotic consumption and is heavily advertised [9]. The increased use of furazidine, often consumed without medical indication, correlates with a 32.8% resistance rate of *E. coli* to nitrofurantoin [9]. Employment status also influences antibiotic use, as working individuals seek quick recovery to return to work [9]. A Polish study found that higher education, having multiple children, and recent antibiotic use are linked to better understanding of antibiotic effectiveness, a trend corroborated by German researchers [34,35]. Overall, the use of antibiotics for both personal and pediatric care appears to enhance knowledge about their efficacy.

In addition to these patient-specific factors, previous studies have also examined factors influencing physicians’ prescribing of antibiotics. A German study reported a high awareness of antimicrobial resistance among general practitioners, which has increased in recent years. However, patient pressure was cited as a self-reported reason for prescribing antibiotics [14]. Other contextual factors pushing physicians towards antibiotic prescribing include frequent visits by the same patient, time pressures, concerns about doctor–patient relationships, legal worries, negative past experiences, an absence of rapid diagnostic tests, and gaps in proper education [12,13].

### 3.2. Evaluation of Knowledge and Attitudes Towards Antibiotics

A Polish study from 2017 indicated that 60% of the general public believed antibiotics could treat viral infections. Yet, awareness seemed to improve between 2009 and 2011, partially due to European Antibiotic Awareness Day campaigns [11]. Despite this, our survey results confirm that antibiotics are often used for conditions typically caused by viruses, even though non-antibiotic treatments are considered effective. In our survey, alternatives to antibiotics were defined as non-antibiotic treatments without further specifics.

Our survey showed a significantly lower number of Polish (21%) and German (17%) respondents who thought antibiotics were effective against viruses compared with earlier studies. This might indicate an improved understanding of appropriate antibiotic use. Nonetheless, our figures for incorrect answers remain high. Unlike previous research, our survey only included patients who had used antibiotics within the last 24 months. This could explain the apparent increase in awareness since recent antibiotic users typically have better knowledge. Noticeably, our results indicate a discrepancy between patients and physicians regarding the stated indications that were treated with antibiotics. For example, patients, much more often than physicians, stated that antibiotics were used for the treatment of rhinitis. In addition to a potential recall bias by patients, this might indicate a knowledge gap of patients regarding indications that require antibiotic treatment. The knowledge gap might be a consequence of a lack of sufficient communication between physician and patient regarding the indication of antibiotics use. A shared decision-making approach, as described in the following section, might be a suitable measure to overcome this gap. It has to be mentioned that the data presented here do not differentiate between patients with one indication and patients with a combination of indications. Therefore, the patient and the physician might have different perceptions of the underlying reason and for which indication the antibiotic has been prescribed. It should be noted that from the portrayed data alone, it is not possible to judge if the antibiotic prescription was appropriate or not.

The survey highlighted that patients acknowledge their role in antibiotic stewardship and exhibit a strong interest in related information. Both physicians and patients appear to agree that patients have a role in reducing antibiotic use, underlining a general willingness amongst patients to be involved in this endeavor and receive relevant information. This underscores the potential impact of targeted information campaigns, particularly emphasizing patient–doctor communication at the point of care where treatment decisions are made.

The perception among Polish and German physicians that better-informed patients can significantly contribute to antibiotic stewardship reinforces the importance of education in this matter. The fact that most patients obtain antibiotics through their doctors, with very few sourcing them elsewhere, suggests a high level of compliance with prescribed antibiotic use.

When discussing expectations for alternatives to antibiotics, most patients and physicians emphasized the need for treatment to prevent the ailment from worsening. This aligns with the common expectation that antibiotics should help prevent symptoms from worsening, as indicated by patients, or aid in averting a decline in the patient’s condition, as mentioned by physicians. A notable contrast was observed among Polish physicians, who placed a high priority (84%) on the idea that alternatives should prevent the worsening of the ailment. In contrast, only 32% of them considered this as crucial regarding antibiotics. Given that viruses are responsible for many respiratory infections, symptomatic therapy is often appropriate, with symptomatic medicines instead of antibiotics serving as suitable treatments. Regarding expectations for antibiotics, German physicians, much more often than their Polish colleagues, stated that they do not want to risk a worsening of the ailment, that they want a reliable and effective therapy, and that an antibiotic is expected to have a quick effect. The underlying reasons for these country differences in the answers cannot be determined with the results presented here but are certainly interesting to address in future studies focusing on structural and cultural differences, such as health policies, access to medications, and healthcare access.

Our results suggests that Polish patients (40%) more often than German patients (19%) assume that antibiotics have no side effects. This might indicate a greater concern for adverse effects among Germans. Furthermore, for German patients (roughly one third), it was much more important that the antibiotic is reimbursed by the health insurance, compared with Polish patients (6%). These different expectations might reflect differences in national reimbursement policies. Regardless of the country, the potential for side effects is a strong rationale for avoiding unjustified antibiotic use. It highlights the importance of reasonable prescribing and considering alternative treatments where they may be effective and have a lower risk profile.

### 3.3. Measures for Reduction of Inappropriate Use of Antibiotics and the Roles of the Key Groups

Physicians are recognized as the critical group for reducing antibiotics usage by themselves, patients, and pharmacists. Consequently, various initiatives addressed to physicians have been implemented to minimize unjustified antibiotic prescribing. The ARena trial (“Sustainable reduction of antibiotic-induced antimicrobial resistance”) is one such initiative that successfully lowered prescription rates and improved adherence to indication-specific guidelines through strategies like e-learning [36]. In line with these findings, physicians acknowledged the need for additional training on antibiotic use [37].

Encouraging careful antibiotic use and shared decision-making between healthcare providers and patients has been recommended and proven to reduce antibiotic consumption [38,39]. Our survey revealed that physicians in Poland and Germany tend to overestimate the number of patients demanding antibiotics and underestimate those who are indifferent. These insights suggest that patients are likely to follow medical advice more than doctors anticipate and that doctor–patient communication has room for enhancement. In particular, a lack of time has previously been cited as the main reason not to talk about antibiotics and the prescription of an antibiotic was described as a measure to avoid confrontation and terminate the consultation. Nevertheless, it has been assumed that physicians attach too much importance on the requests of their patients regarding antibiotics [14]. This fits to the results of our presented survey which show that physicians overestimate the proportion of patients demanding antibiotics. Improved communication about the patient’s expectations might be a way to resolve this discrepancy. Emphasizing symptomatic treatments, when appropriate, is recommended, especially within a shared decision-making framework.

When considering symptom-relieving therapies, non-antibiotic alternatives play a vital role and are a critical factor in joint treatment decisions. For example, interaction with *E. coli* makes D-mannose an effective non-antibiotic option for uncomplicated lower urinary tract infections [40]. In the case of respiratory infections, non-antibiotic therapies like octenidine dihydrochloride and cineole can provide symptom relief, meeting an important need emphasized by patients in this survey [30,41]. Cineole, in particular, has been effective in the treatment of acute inflammatory disorders of the respiratory system, such as the common cold, bronchitis, and rhinosinusitis [42,43,44].

Moreover, the C-reactive protein (CRP) value is considered one of several key factors when deciding whether to prescribe antibiotics, which corresponds with practitioners’ observations that patients often desire quantitative tests to verify the necessity of an antibiotic [28,45]. This is consistent with our findings that physicians view rapid test results for bacterial or viral infections as crucial to reducing antibiotic prescriptions. The CRP levels can be used to differentiate bacterial from viral infections since CRP is more elevated in the case of bacterial infections [46]. Point-of-care (POC) testing of CRP has already been implemented, at least to some extent, in several European countries, Germany and Poland among them [47,48]. In primary care in Germany, however, the reimbursement of POC CRP tests is low [49]. Therefore, these tests are usually not applied. A survey in pharmacies regarding POC CRP testing in countries where this has been implemented, such as Poland, has revealed barriers for the use of these POC CRP tests. These were, for example, time constraints, heavy documentation, and remuneration issues [48].

Previously recommended measures such as “delayed prescription” are designed to minimize unjustified antibiotic use [28]. In our survey, 20% of antibiotic recipients in Germany and 25% in Poland indicated that their doctor gave them antibiotics as a preventive measure. However, it was unclear if these instances involved “delayed prescriptions”. Since not all “preventive” antibiotics were likely dispensed in this manner, there is potential for better practice in this area. Despite only moderate support for delayed prescriptions in our survey, employing this strategy along with other measures might help decrease antibiotic utilization, mainly because it is relatively straightforward to implement. Patients may receive antibiotic prescriptions during an initial visit if they face long waiting times for medical appointments so that they can purchase antibiotics when they feel it is necessary. Physicians have reported that the practice of delayed prescriptions help as a “social tool” to negotiate treatment decisions with patients and use this as an opportunity to educate patients on the use of antibiotics in general. However, it has to be mentioned that there was some ambiguity about when and how delayed prescriptions should be used in general practice and in which situations they are beneficial [28]. Therefore, this practice suggests an area where further improvement is possible in reducing antibiotic use.

The WHO Europe highlights that pharmacists are pivotal in promoting appropriate antibiotic use and combating AMR [50]. They can contribute by suggesting early non-antibiotic options for symptom relief. Nevertheless, our findings suggest pharmacists perceive their responsibility for antibiotic stewardship as limited and show a moderate willingness to engage in these efforts actively. This may be partly due to the obligation pharmacists have to dispense medications as prescribed by a physician.

Pharmacists become particularly crucial when they are the initial point of contact for patients with mild symptoms, since here, early symptomatic treatment can be recommended, or during seasonal epidemics with significantly higher patient volumes. In addition, patients with moderate symptoms first visit the pharmacy before doctoral consultation occurs. In these cases, the pharmacy also plays an important role for initial advice. Looking ahead, it is essential to reinforce pharmacists’ significant role in recommending appropriate symptom-relief options, like cineole for treating rhinosinusitis, acute bronchitis, or the common cold to circumvent unjustified antibiotic prescriptions [42,43,44]. This is particularly the case when the positive effect of preventing an escalation of the common cold supports antibiotic stewardship, as the recent work of Michalsen and colleagues concluded [44]. Here, the pharmacist is the earliest point of intervention. This effort could be supported by well-informed patients and educational materials, empowering pharmacists in their guidance.

Thus, strategies to encourage the prudent use of antibiotics must continuously be evaluated and implemented, including physicians, pharmacists, and patients.

### 3.4. Limitations of the Work

Here, challenges in assessing ailments leading to the prescription and use of antibiotics should be mentioned. As usual, in the case of self-completion retrospective questionnaires, a recruitment bias and the fact that patients answered the questionnaire from their memory are potential limitations. This factor contributed to a recall bias, causing discrepancies between patients and doctors in the reasons given for antibiotic use, since patients might not have been able to correctly recall the indication for which the antibiotic has been prescribed. However, respondents were recruited according to national representative structure (sex, age, region, income, education) to minimize a selection bias and were not informed beforehand on the purpose of this survey. Since the aim of this study was the evaluation of personal attitudes and knowledge, the presented results are solely based on answers to the questionnaire without analysis of data from other sources. Additionally, it should be mentioned that the terminology of the questionnaires was adjusted for patients to allow participants with less knowledge of medical terms to complete the survey. Furthermore, the original questionnaires were provided in German and Polish, while this manuscript is written in English, so minor inaccuracies in the course of translation cannot be ruled out completely, given that the use of words might be different in the respective languages.

## 4. Materials and Methods

Online questionnaires were prepared for physicians, pharmacists, and patients in Germany and Poland with the overarching aim to assess drivers for the (unjustified) use of antibiotics for respiratory diseases. Importantly, only antibiotic treatment of indications without a bacterial cause is considered avoidable and unjustified. Patients in Germany answered the questionnaire on 18 August 2021, and in Poland on 6 September 2021. Patients were recruited until 1000 participants per country (Germany and Poland, respectively) were included. Healthcare professionals answered the questionnaire from 23 to 30 September 2021. The questionnaire was a self-completion online questionnaire on a platform from the service provider Ipsos (Paris, France).

### 4.1. Participants

The present survey was conducted with participants from the three target groups: physicians (GPs, PEDs, ENTs), pharmacists, and patients in Germany and Poland (Table 1). Participants were members of an online-access-panel and declared their willingness to participate in a survey. Recruitment occurred via banners on (specialist) portals and a representative, structured sample of participants was assembled.

### 4.2. Survey Questionnaire

The presented data are based on online questionnaires with 15–20 questions. Three individual questionnaires were applied in German and Polish, one for each group (physicians, pharmacists, and patients). The time required for the self-completion interview was between 10 and 15 min. Ipsos conducted the interview and the following data analysis and data quality checks. The first part included demographic questions. For questionnaire design and data analysis, extensive quality assurance measures were followed by the service provider Ipsos. In order to ensure the reliability and consistency of the data, a pretest via a soft launch of the questionnaire was conducted. Incoming results of the first days of fieldwork were closely monitored. The survey was conducted in adherence to the § 24 ‘IHK/ESOMAR Code for the Practice of Market and Social Research’, which outlines rigorous ethical and methodological standards for conducting market and social research. The survey was also conducted in accordance with the recommendations and terms of business of the ADM (Arbeitskreis Deutscher Markt- und Sozialforschungsinstitute e.V.), further ensuring that industry-standard measures were followed to maintain the integrity of the data collected.

For physicians, these were specialty, age, duration of professional conduct, rural or urban location, type of practice, and whether mainly patients with private or statutory health insurance are treated. The participant was screened out if the physician had a profession other than general medicine, gynecology, pediatrics, internal medicine, ENT, or if the profession was practiced for less than one year.

For pharmacists, these demographic questions were about function in the pharmacy, duration of professional conduct, and percentage of working time used for over-the-counter sales. The participant was screened out if the pharmacist had a duration of professional conduct of two years or less or spent less than 70% of working time on over-the-counter sales.

For patients, the demographic questions were about gender, age, zip code, use of antibiotics in the last 24 months, marital status, number of kids up to 14 years in the same household, and type of health insurance. The interview was continued if the patient received an antibiotic in the last 24 months. Otherwise, it was finished at that point.

The specific content-related questions can be found in the descriptions of the respective figures and tables in Section 2. Here, it should be noted that there was a translation bias considering the term “influenza-like infection”. The German questionnaire’s name was “grippaler Infekt” which roughly translates to “influenza-like infection”. The Polish questionnaire asked for “grypa”, which is influenza in the narrower sense. Therefore, the answer “influenza-like infection” was not included in Figure 2 and Figure 3.

### 4.3. Data Analysis

Usually, the answers for the three physicians’ specialties were summarized and are shown as average physician results. In cases where the results of the three specialties were particularly interesting, each of the three specialty results are shown (Figure 3). The group of pharmacists also included pharmacy technicians. In general, the numbers of responses are presented as a percentage of the respective subgroup. Where indicated, the percentages were calculated from top-box rankings with the indicated number of top-boxes. Data analysis was provided by the service provider Ipsos. No statistical analysis has been carried out since the design of this survey with multiple choice answers does not qualify for such an analysis.

## 5. Conclusions

Antibiotic resistance is a major challenge for public health. To reduce the frequent unjustified use in outpatient care, it is important to identify drivers for antibiotics prescription. Therefore, we surveyed physicians, patients, and pharmacists regarding factors related to ambulant antibiotics prescriptions. Furthermore, knowledge and expectations about antibiotics were assessed, showing that patients desire symptomatic treatment and fast effect.

Interestingly, physicians generally underestimate the fraction of patients indifferent towards antibiotic treatment. Pharmacists and physicians acknowledge different measures for reducing antibiotics consumption, showing a general interest in alternative treatments, which should primarily provide symptom relief. Generally, patients are interested in information on antibiotics and healthcare professionals are willing to contribute to avoid the use of antibiotics. However, pharmacists believe that they do not contribute significantly to avoiding unjustified antibiotic use, indicating that they might underestimate their role in advising on early treatment. Still, in order to harmonize the expectations of patients and healthcare professionals, confidential dialogues with patients can be an important starting point. In order to achieve this goal, we conclude that there is a need for better information about effective alternatives to improve patient’s symptoms in case of virus-induced ailments. Additionally, the improved training of healthcare professionals, relieving time pressure, shared decision-making, overcoming barriers for POC testing, and possibly delayed prescriptions are measures that might contribute to lowering the numbers of unjustified antibiotic prescriptions.

## Figures and Tables

**Table 1:** Overview of the participants sorted by country and role. In interviews for pediatricians, the questions were adapted for the parents’ view. Physicians were asked about their number of patients in an average week. Results were for Germany, an average of 178 patients/week and for Poland, an average 110 patients/week.

	Germany	Poland
Physicians (Total)	181	146
Physicians (GPs)	61	60
Physicians (Pediatricians)	60	60
Physicians (ENTs)	60	26
Pharmacists	142	134
Patients (Total)	1000	1000
Receivers of antibiotics in the past 24 months	359	508

**Table 2:** Source of antibiotics. Question: Patients: Think of the last time you took antibiotics—where did you get them from (multiple answers possible)? (DE, n = 359; PL, n = 508 are the bases for further evaluations, being the patients who received antibiotics).

Source of Antibiotics (AB) for Patients	Germany (DE) (n = 359)	Poland (PL) (n = 508)
From doctor on prescription for immediate use	60%	69%
From the doctor as a precaution	20%	25%
Own medicine cabinet	8%	6%
Without a prescription from somewhere else	6%	2%

**Table 3:** Knowledge about antibiotics. Questions: Patients: In your opinion, which statements apply to antibiotics (multiple answers possible)? Physicians: What statements do you think apply to antibiotics (multiple answers possible)?

Agreement with Statements About (%)	Patients DE (n = 359)	Patients PL (n = 508)	Physicians DE (n = 181)	Physicians PL (n = 146)
Antibiotics are effective against bacteria	45%	42%	96%	96%
Antibiotics are effective against viruses	17%	21%	1%	1%
Antibiotics always help against flu and colds	13%	16%	1%	0%
For urinary tract infections (UTIs)/cystitis, only antibiotics help	18%	11%	8%	15%
Sometimes being the only effective solution	49%	57%	74%	84%
Taking antibiotics increases the risk that the disease-causing germs will become more resistant	35%	23%	71%	81%
Antibiotics often have side effects such as diarrhea, allergic reactions, or impairment of the intestinal flora	40%	17%	72%	75%
Have side effects for the duration of treatment	21%	25%	38%	47%
Have long-term side effects even after ingestion	16%	13%	23%	14%
Do not know	3%	4%	0%	0%

**Table 4:** Agreement of pharmacists to measures for reducing AB use. Questions: Pharmacists: (1) When you think about advising patients who come to you with an antibiotic prescription: How important is it to you personally to address the following topics (scale of importance 1–5)? Represented here is the proportion of answers on the two categories with the highest importance (Top-2 boxes). (2) What would help you to influence further reduction of antibiotics (multiple answers possible)? Results for the answer “Legally secured self-dispensing of antibiotics” (agreement DE: 11%, PL 4%) not shown here.

Importance to Address Topic with Customer (Top-2 Boxes, Agreement is High or Very High in %)	Pharmacists DE (n = 142)	Pharmacists PL (n = 134)
Frequency and duration of use	93%	87%
Advice on interactions with other medications	89%	66%
Supportive therapy options to alleviate or prevent side effects (e.g., diarrhea)	85%	84%
Advice on side effects of antibiotics	80%	66%
Advice on the general reduction of antibiotics to the necessary minimum	44%	45%
Advice on alternative products to antibiotics	39%	30%
**Helpful measures to reduce AB (% of responders agreeing)** **(without Top-box grouping)**		
A rapid test result for bacterial or viral findings	54%	81%
Delayed prescription	37%	40%
Suitable alternatives to antibiotics	58%	35%
More intensive consultation of patients	39%	42%
A counseling tool for the most common indications where there are alternatives	52%	29%

**Table 5:** Agreement of physicians to measures for reducing AB use. Question: Physicians: What would help you to further reduce the prescription of antibiotics (scale of importance 1–5)? Represented here is the proportion of answers in the two categories with the highest importance (Top boxes).

What Would Help You to Further Reduce the Prescription of Antibiotics? (Top-2 Boxes, Agreement is “High” or “Very High” in %)	Physicians (DE)	Physicians (PL)
More time to advise patients	69%	71%
A rapid test result for bacterial or viral findings	77%	90%
Routine test in the laboratory as to whether bacteria or viruses are causal	45%	73%
Suitable products alternative to antibiotics	58%	63%
A counseling tool for the most common indications where there are alternatives	53%	67%
More frequent use of the so-called delayed prescription	46%	37%
Better remuneration for ’talking medicine’	70%	33%
Better cooperation with pharmacies	26%	29%
Better informed patients	73%	82%
Note to patients on Green Prescription/reimbursement of alternative products	41%	n.a.
Medical guidelines that include more alternative ingredients and therapies	63%	86%
Clinical studies demonstrating the suitability of non-antibiotic therapies	76%	86%

## Data Availability

All relevant data are reported within this article and its appendices. Anonymized, aggregated data tables reflecting the results of the data used herein for the relevant questions can be made available upon reasonable request. Data Source is © Ipsos|antimicrobial resistance (AMR) Driver Analysis, Poland and Germany 2021.

## Supplementary Materials

The following supporting information can be downloaded at: https://www.mdpi.com/article/10.3390/antibiotics13121188/s1, Figure S1: Physician-reported antibiotic prescriptions per week; Figure S2: Diagnostic behavior towards AB prescriptions
