# Supplementary material for: Antibiotics Usage and Avoidance in Germany and Poland: Attitudes and Knowledge of Patients, Physicians, and Pharmacists
Source: Antibiotics (Basel). 2024 Dec 6;13(12):1188. doi: 10.3390/antibiotics13121188 (PMC11672592; doi:10.3390/antibiotics13121188)
Supplement: Supplementary file 1 [file antibiotics-13-01188-s001.zip › antibiotics-3227929-supplementary.pdf]

antibiotics

MDPI

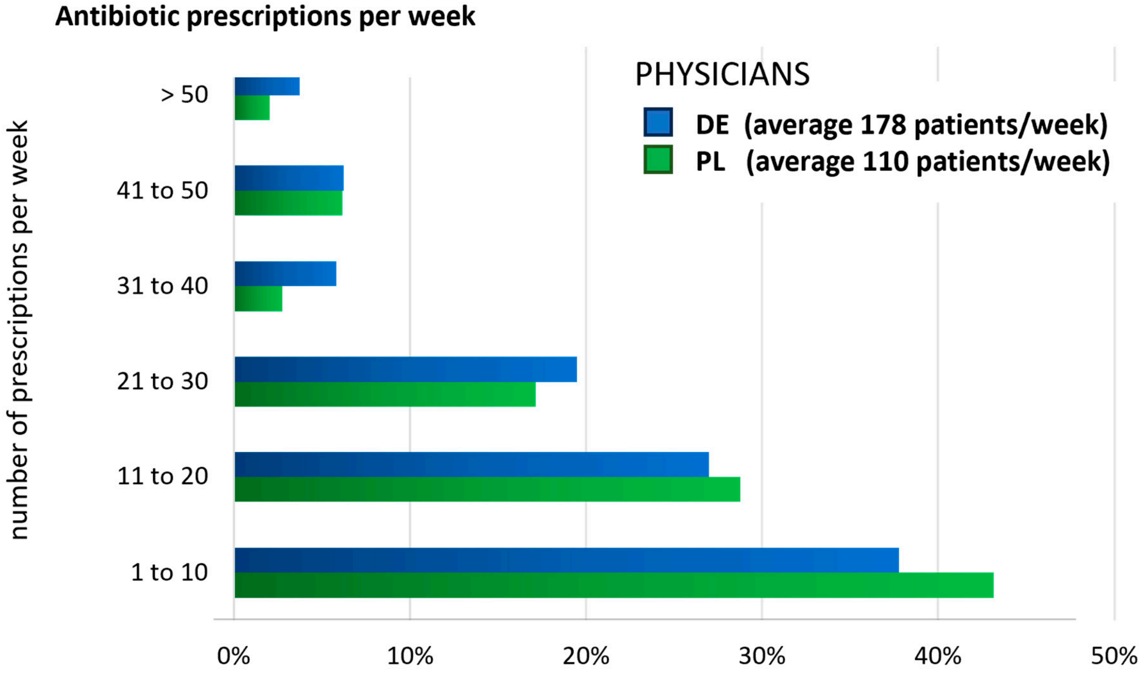

Supplementary Figure S1. Physician-reported antibiotic prescriptions per week. Physicians were asked about the number of patients and number of antibiotic prescriptions in an average week. Questions: Physicians: How many patients do you see in your practice in an average week (results indicated in the top right corner)? How many patients do you prescribe antibiotics for in an average week (results indicated in the bar diagram)?

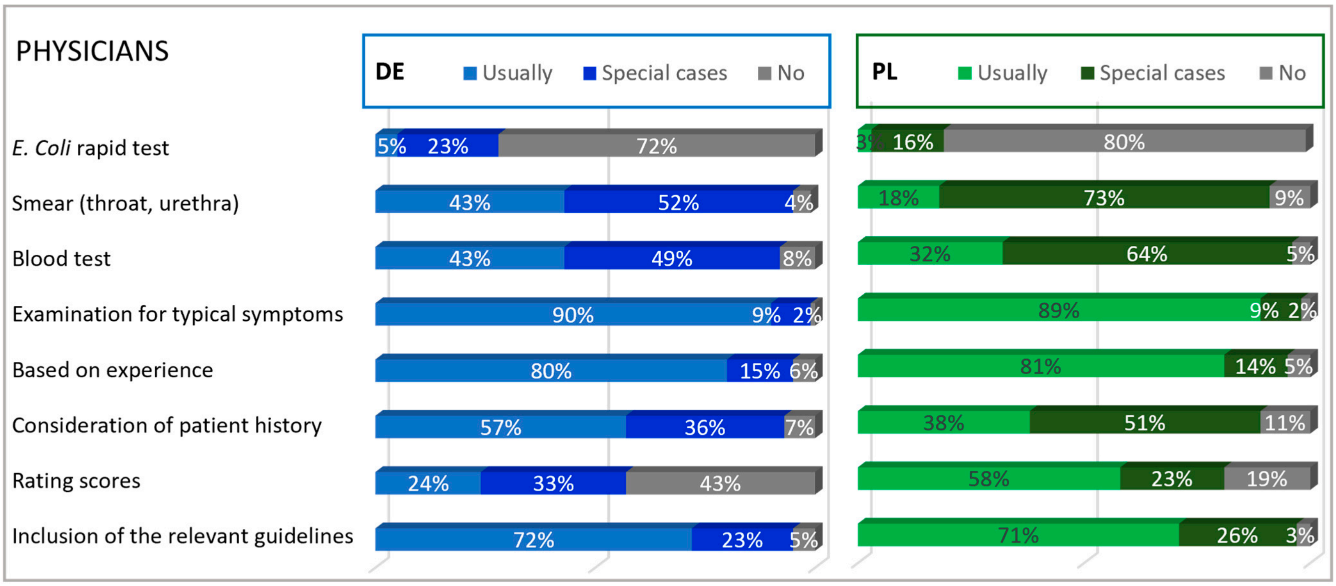

Supplementary Figure S2. Diagnostic behavior towards AB prescriptions. Questions: Physicians: How do you proceed to secure an antibiotic prescription? Please tick all relevant measures. Then we are interested in which ones may only be used in exceptional cases and which you never use. Rounding effects cause slight deviations from 100% but have no relevant impact on the general results.
